# Case Report: Early infantile drug-resistant epilepsy and gastrointestinal dysmotility associated with a *de novo GNAO1* variant and a 16p13.11 microdeletion

**DOI:** 10.3389/fped.2026.1873389

**Published:** 2026-08-28

**Authors:** Xuan Liu, Rong Luo, Jianjun Wang, Xiaolu Chen, Yao Deng

**Affiliations:** 1 Department of Pediatrics, West China Second University Hospital, Sichuan University, Chengdu, Sichuan, China; 2 Key Laboratory of Obstetrics & Gynecologic and Pediatric Diseases and Birth Defects of the Ministry of Education, Sichuan University, Chengdu, Sichuan, China

**Keywords:** 16p13.11 microdeletion, developmental epileptic encephalopathy, gastrointestinal dysmotility, *GNAO1* gene, patent ductus arteriosus

## Abstract

**Background:**

The *GNAO1* gene is located on the long arm of chromosome 16 (16q13) and is associated with Developmental and Epileptic Encephalopathy 17. It encodes a protein involved in regulating various neurotransmitters and neuronal growth and development. The 16p13.11 microdeletion is a genomic deletion in the p13.11 region of the short arm of chromosome 16, previously reported to predispose individuals to neurodevelopmental disorders. These two regions do not overlap on the chromosome, and the 16p13.11 microdeletion does not involve the *GNAO1* gene locus.

**Case presentation:**

We reports a 1-month-old infant presenting with concurrent *GNAO1 (p. G203R)* gene variation and a 16p13.11 microdeletion. The patient initially presented with recurrent abdominal distension in the neonatal period, which gradually progressed to include focal seizures and intractable epileptic spasms. Interictal EEG showed multifocal discharges and burst-suppression patterns. Brain MRI revealed no abnormalities. A full gastrointestinal series suggested intestinal obstruction and gastro-esophageal reflux. Improved whole-exome sequencing identified: 1) A single nucleotide variantion: *GNAO1, NM_020988.3:exon6: c.607G* *>* *A (p.Gly203Arg)*. 2) A copy number variation: A pathogenic 1.453 Mb deletion in the 16p13.11 region.

**Conclusions:**

We describe the developmental trajectory of an infant with overlapping features of gastrointestinal symptoms and drug-resistant epilepsy, carrying a *GNAO1* variant and a 16p13.11 microdeletion. This case provides insights into the contribution of both single nucleotide variant and copy number variant to the phenotype.

## Background

Developmental epileptic encephalopathies (DEEs) are a group of severe neurodevelopmental disorders characterized by early-onset intractable seizures and intellectual disability of variable degree or developmental impairment (1). Genetic causes account for a very high proportion of DEEs, primarily in the form of single nucleotide variant and chromosomal copy number variants (CNVs). In 2013, Nakamura et al. (2) first reported *GNAO1* variants in patients with DEE17, characterized by intractable seizures beginning in the first week of life and an EEG showing a burst-suppression pattern. In 2016, Kulkarni et al. (3) described a severe, progressive *GNAO1*-related movement disorder phenotype devoid of epilepsy. In recent years, accumulating evidence has confirmed that pathogenic variants in the *GNAO1* gene can lead to a spectrum of *GNAO1*-related disorders characterized primarily by epilepsy, movement disorders, and neurodevelopmental delay. Currently, more than 400 germline *GNAO1* mutations have been identified across multiple genomic databases and reported *GNAO1* patients, including missense and nonsense mutations, insertions/deletions, and splice-affecting variants. Among these, over 80 pathogenic *GNAO1* variants (mostly missense) have been characterized, and the three codons *Gly203, Arg209, and Glu246* are hot spots for disease mutations (*databases.lovd.nl/shared/genes/GNAO1;ClinVar:* http://www.ncbi.nlm.nih.55gov/clinvar/?term=gnao1%5Bgene%5D&redir=gene).

CNVs are well-established genetic causes of epilepsy. Specifically, within DEEs, *de novo* pathogenic CNVs are identified in about 5% to 10% of patients (4). The 16p13.11 microdeletion is a rare CNV. It is associated with a syndrome characterized by motor developmental delay, microcephaly, gastroesophageal reflux disease, and congenital heart defects (5). Commonly reported 16p13.11 microdeletion spans 0.8–3.3 Mb. This region encompasses several core protein-coding genes, including the *NDE1* gene (encoding a neurodevelopmental protein) and the *MYH11* gene (encoding the major contractile protein of smooth muscle cells). Both genes have been highlighted as key disease-causing contributors in multiple studies (5).

## Case report

A 1-month-old female infant, born via full-term cesarean section to healthy, non-consanguineous parents. The mother had polyhydramnios at 28 weeks of gestation. She experienced mild perinatal asphyxia. Delayed meconium passage preceded recurrent abdominal distension. At age 2 weeks, unprovoked focal seizures manifested as repetitive blinking, facial twitching, occasionally accompanied by limb jerks; duration 10 s to 2 min; duration 10s–2 min; variable frequency. Around 3 weeks of age, clusters of subtle head nodding and bilateral upper limb elevation movements appeared daily. Concurrently, abdominal distension became progressively more noticeable, exacerbated by crying. Physical examination demonstrated marked abdominal distension with tympanic percussion note. Because of the patient's irritability, assessment of limb tone and strength was limited.

**Figure 1:**
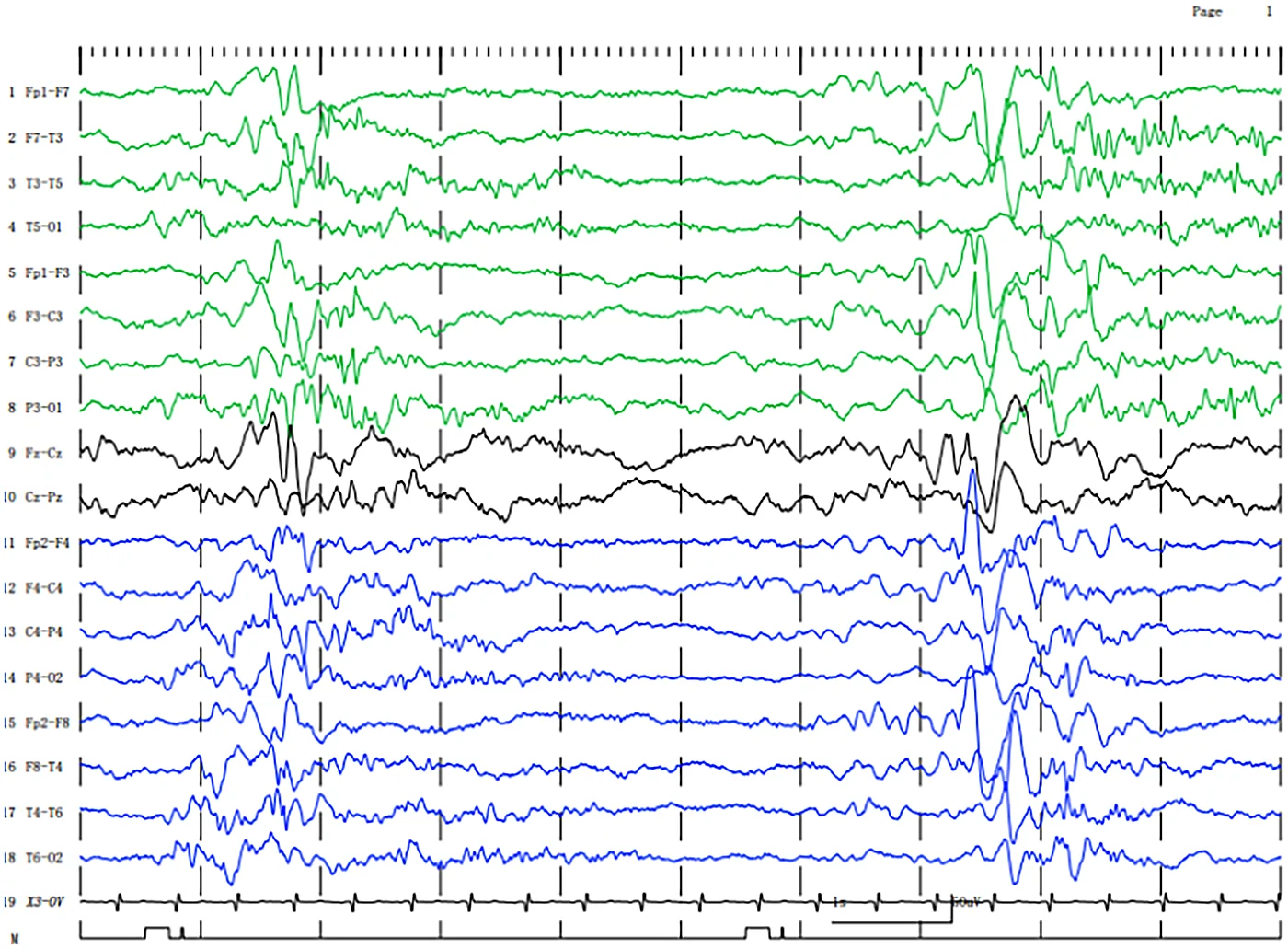
Video EEG recording. Burst-suppression pattern recorded at 1 month of age before ACTH therapy.

**Figure 2:**
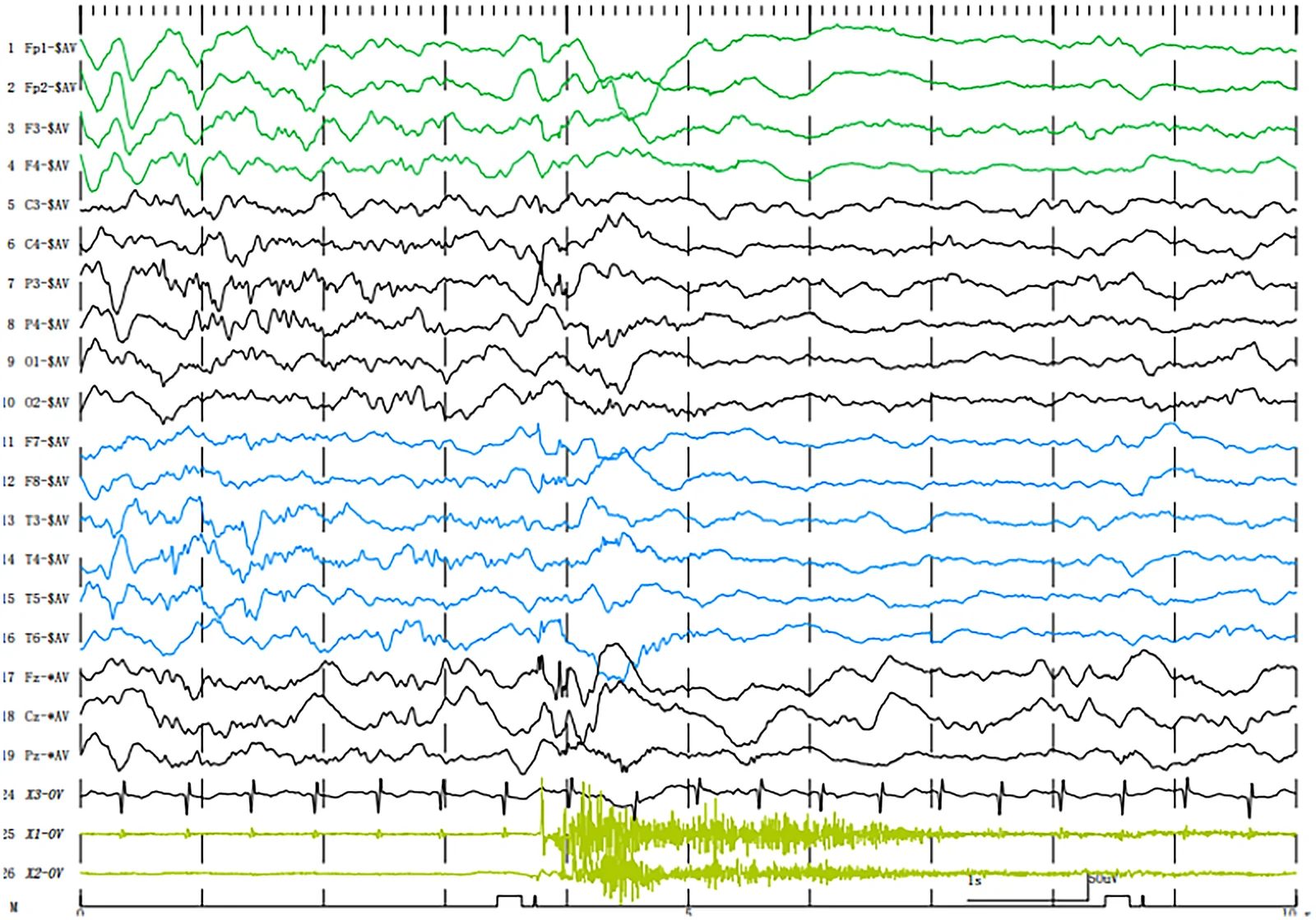
Ictal EEG confirming epileptic spasms.

**Figure 3:**
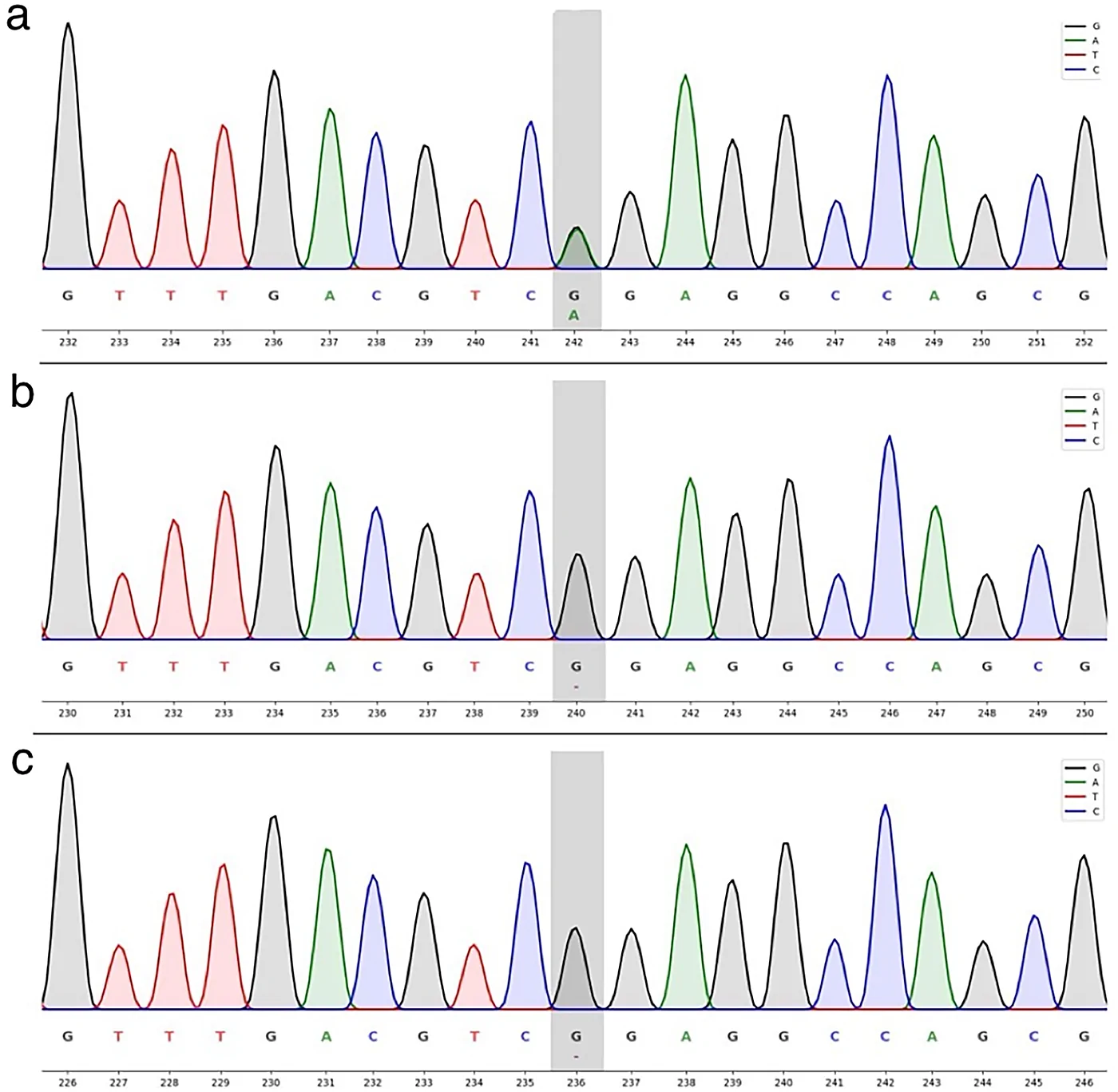
Genetic analysis results of the infant and her parents. **(a)** The infant; **(b)** the infant of mother; **(c)** the infant of father. A novel heterozygous variant *(exon6:c.607G* *>* *A)* in the *GNAO1 gene (NM_020988.3)* was detected in the infant. No mutation in the *GNAO1* gene was detected in her parents.

She had a normal laboratory evaluation, a normal brain MRI/ MRA and Renal and bladder ultrasound. Video-EEG at age 1 month revealed a burst-suppression pattern during the interictal period (Figure 1). Ictal recordings confirmed epileptic spasms (Figure 2). Echocardiography: patent ductus arteriosus (2 mm) and patent foramen ovale (1.5 mm). Superficial ultrasound: right inguinal hernia. Upper gastrointestinal series with small bowel follow-through demonstrated: (1) Dilated bowel loops, suggestive of possible intestinal obstruction, (2) Gastroesophageal reflux. Improved whole-exome sequencing identified (Figure 3): (1) A single nucleotide variantion: *GNAO1, NM_020988.3:exon6: c.607G* *>* *A (p.Gly203Arg)*. This *de novo* variant (maternal: wild-type; paternal: suspected mosaicism). According to the ACMG guidelines, the c.607G > A variant was classified as pathogenic, PS2_VeryStrong: The variant was confirmed to be *de novo* in the proband (with the possibility of parental germline or somatic mosaicism not excluded). (2) A copy number variation: A pathogenic 1.453 Mb deletion in the 16p13.11 region (chr16:14,779,387–16,232,851). Paternally inherited (father carries a 1.411-Mb deletion at chr16:14,833,832–16,245,180). This deletion overlaps a known haploinsufficient region (chr16:15,417,854–16,198,408; HI = 2) and corresponds to the pathogenic ClinVar variant 58732, which is associated with developmental delay and seizures.

**Figure 4:**
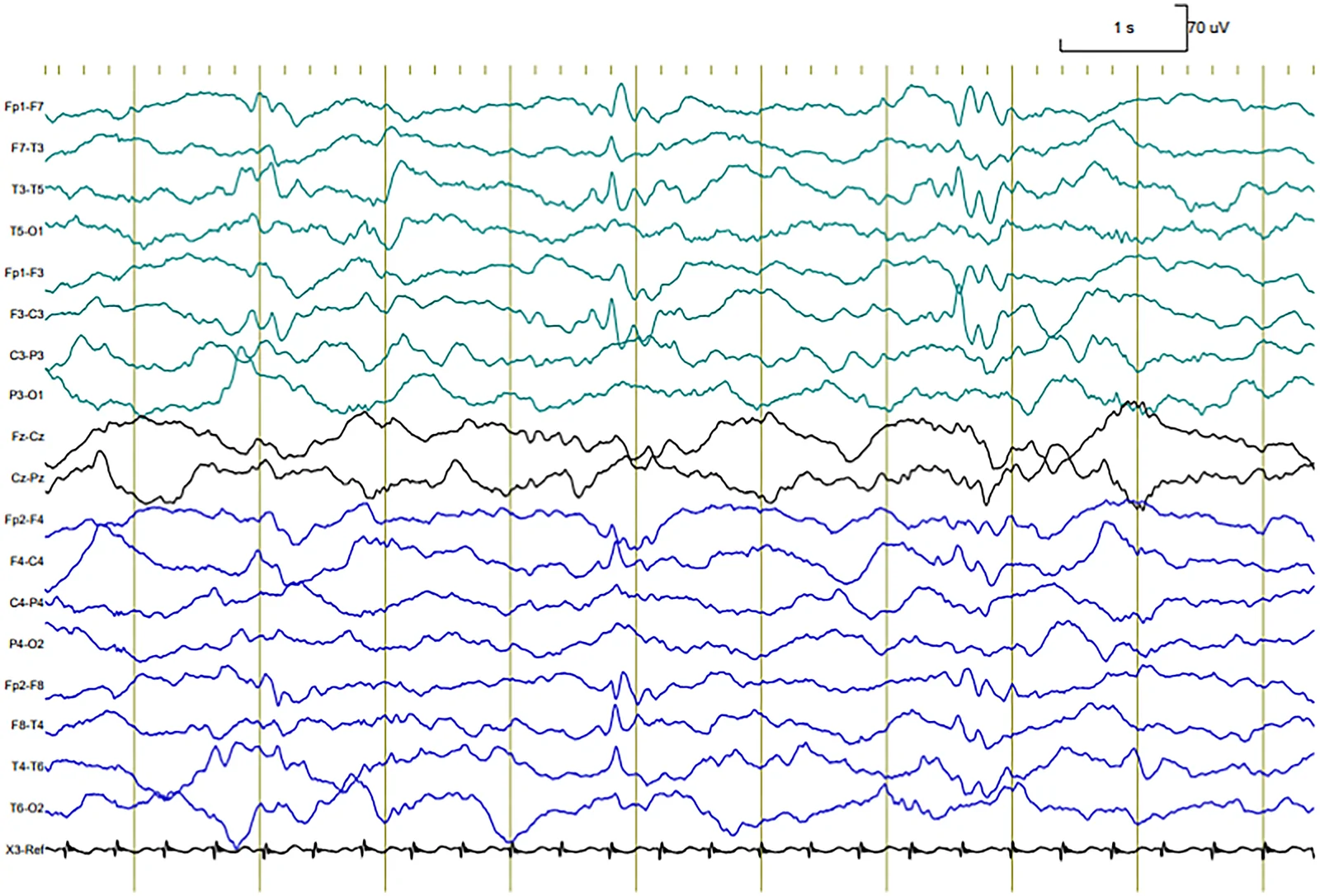
Persistent multifocal sharp waves, predominantly during sleep, at 2 months of age under ACTH and LEV therapy.

The infant developed seizures in the neonatal period. Treatment with levetiracetam and subsequently adrenocorticotropic hormone was initiated, but seizure control remained suboptimal. At 2 months of age, video-EEG continued to show predominantly multifocal discharges (Figure 4), and epileptic spasms were captured. Due to medically refractory abdominal distension, the infant underwent exploratory laparotomy, which revealed ileal stenosis, prompting resection of the stenotic segment and ileostomy. Histopathology of the specimen showed scattered immature ganglion cells in the submucosa. Following the surgery, the abdominal distension resolved, enteral feeding was resumed, and bowel movements normalized. At 4 months of age, valproate was added. Initially, seizure control was good, the infant's development was acceptable, and the infant could briefly hold the head up. However, focal seizures subsequently recurred. Currently 8 months old, the infant exhibits global developmental delay, including lack of head control and inability to sit unsupported. Neurological examination reveals normal muscle tone and absence of overt dystonia. The antiepileptic regimen was titrated to valproate and oxcarbazepine. However, seizures remained uncontrolled at the target dose; subtherapeutic valproate levels and ileostomy prolapse were noted. Suspected drug leakage through the stoma prompted surgical re-evaluation. Additionally, the infant was found to have a non-incarcerated inguinal hernia at over 2 months of age. At 6 months, hypothyroidism was diagnosed, and levothyroxine sodium replacement therapy was initiated.

## Discussion

We report a female infant harboring concurrent pathogenic variants in the *GNAO1* gene and a 16p13.11 microdeletion, which were directly linked to her complex phenotype primarily characterized by DEE17, functional gastrointestinal obstruction, patent ductus arteriosus, gastroesophageal reflux, inguinal hernia, and hypothyroidism. This infant presented with prominent clinical manifestations since the neonatal period. Although each variant has been reported separately, this is the first reported case harboring both.

The *GNAO1* gene is located on the long arm of chromosome 16 (16q13) and encodes the alpha subunit of the guanine nucleotide-binding protein Go (G*α*o). G*α*o couples to important G protein-coupled receptors, including GABA_B receptors. At the molecular level, *GNAO1* variants are broadly classified into gain-of-function (GOF) and loss-of-function (LOF) types. Typically, GOF mutations associate with movement disorders, whereas LOF mutations link to epilepsy (6). This cellular distinction manifests as follows: variants exhibiting reduced plasma membrane localization and significantly impaired G*βγ* binding are primarily associated with DEE. In contrast, variants with normal localization to the plasma membrane and Golgi apparatus typically correlate with the neurodevelopmental disorder with involuntary movements phenotype (7). However, *p.G203R* represents a notable exception. Molecularly defined as a GOF mutation with normal plasma membrane and Golgi localization—features typically predictive of the neurodevelopmental disorder with involuntary movements—it nevertheless paradoxically manifests in most patients as a complex phenotype encompassing both epilepsy and movement disorders (2, 8). Currently, *p.G203R* is a recurrent hotspot variant in the *GNAO1* gene. Previous studies report that the majority of patients with this variant develop DEE within 92 days after birth, which progresses to severe movement disorders within the first two years of life (9). In this case, the infant presented with a more prominent DEE phenotype, while movement disorders were less apparent. This phenotypic divergence likely reflects the patient's young age, as the patient is currently only 8 months old and motor symptoms may not yet be fully manifest. Therefore, long-term follow-up is warranted.

In patients with the *p.G203R* variant, observed DEE subtypes include early infantile DEE (EIDEE), epilepsy of infancy with migrating focal seizures (EIMFS), and infantile epileptic spasms syndrome (IESS). No unifying EEG pattern has been described; rather features are concordant with epilepsy phenotype. Brain MRI is often normal or shows nonspecific findings, such as diffuse cerebral atrophy and corpus callosum thinning (9, 10). The spectrum of variants is predominantly composed of heterozygous missense mutations, though in-frame deletions and splice-site mutations have also been reported. While most occur *de novo*, parental mosaicism has been documented in multiple individuals (11, 12). The clinical presentation in our patient—DEE with a burst-suppression pattern on EEG and a normal MRI—is consistent with the features described above. The paternal origin of the *GNAO1* variant is suspected to involve mosaicism (4|120 reads), indicating possible low-level mosaicism. Further studies to confirm and characterize this mosaicism were not pursued due to parental preference. A sex effect with female predominance has been proposed in *GNAO1*-related disorders. Specifically, patients harboring the p.G203R variant exhibit severe phenotypes with a female bias, a phenomenon potentially attributable to more severe, possibly non-viable manifestations in male carriers (9). This view is further supported by studies reporting similar gender-based phenotypic differences in *GNAO1* mutant mouse and Drosophila models (13, 14). Our patient, a female with the *p.G203R* variant who presented with a severe phenotype, is consistent with this observation.

The 16p13.11 microdeletion is a genomic copy number variant characterized by phenotypic heterogeneity and incomplete penetrance. This region can be divided into three single-copy sequence intervals, among which interval II (chr16:15,417,854–16,198,408) represents the critical region for genomic variation. The infant carried an approximately 1.453 Mb deletion at 16p13.11 (chr16:14,779,387–16,232,851), and her father carried an approximately 1.411 Mb deletion at 16p13.11 (chr16:14,833,832–16,245,180). The two deletions are highly overlapping (>1.39 Mb) and both fully cover the critical region noted above. The minor differences in the boundary coordinates (approximately 54 kb at the left end and 12 kb at the right end) arise from technical breakpoint estimation error due to probe coverage limitations of whole-exome sequencing-based CNV detection, rather than representing distinct deletion events. The deleted region substantially overlaps with haploinsufficiency-sensitive regions/genes, suggesting that the pathogenic mechanism primarily involves haploinsufficiency of the relevant genes. The typical 16p13.11 deletion encompasses multiple protein-coding genes, among which *NDE1, MYH11, ABCC1,* and *ABCC6* are the most commonly studied and are closely associated with neurodevelopment and various systemic disorders (15). The protein encoded by *NDE1* (OMIM: 609449) forms a complex with LIS1 and dynein to regulate microtubule organization and neuronal migration. Its haploinsufficiency is considered a key factor leading to epilepsy and neurocognitive impairment (16). The *MYH11*(OMIM: 160745) gene encodes the smooth muscle myosin heavy chain. Its deletion or dysfunction impairs contractile function in vascular and gastrointestinal smooth muscle, and is linked to hereditary thoracic aortic disease, patent ductus arteriosus, and severe intestinal dysmotility such as Megacystis Microcolon Intestinal Hypoperistalsis Syndrome (17). Both *ABCC1* (OMIM: 158343) and *ABCC6* (OMIM: 603234) belong to the ATP-binding cassette transporter superfamily, encoding multidrug resistance proteins 1 and 6, respectively. Evidence suggests that autosomal dominant deafness is caused by heterozygous mutations in *ABCC1* on chromosome 16p13.11 (18). Deletion of the 16p13.11 region containing this gene leads to global developmental impairment, including speech delay, learning difficulties, and hearing impairment (19). The function of *ABCC6* remains unclear, but its variants may be associated with cardiovascular injury and neuropsychiatric abnormalities (20). The epilepsy, developmental impairment, functional gastrointestinal obstruction, patent ductus arteriosus, gastroesophageal reflux, and inguinal hernia observed in this case are likely closely related to haploinsufficiency of the genes mentioned above. Furthermore, this microdeletion exhibits low penetrance (approximately 13.1%) and a broad phenotypic spectrum, including but not limited to developmental delay, intellectual disability, autism, multiple congenital anomalies, cardiovascular abnormalities, epilepsy, and gastrointestinal abnormalities. Some carriers may present with mild phenotypes or be unaffected (21, 22). In this case, the deletion was inherited from the patient's phenotypically normal father, which is consistent with its incomplete penetrance. Prenatally, fetuses with a 16p13.11 microdeletion often have unremarkable ultrasounds, with nonspecific findings occasionally including cardiovascular anomalies or ventriculomegaly (15, 23). In this case, prenatal evaluation only revealed polyhydramnios. This observation highlights the need for further cases to strengthen prenatal genotype-phenotype correlations for this microdeletion.

Although the 16p13.11 microdeletion does not involve the *GNAO1* gene (located at 16q13), it causes haploinsufficiency of regionally encoded genes such as *NDE1*. *NDE1*, a component of the Lis1/dynein complex, mediates microtubule-dependent neuronal migration and synaptic vesicle transport; its reduced expression disrupts synaptic homeostasis and lowers seizure threshold. *GNAO1* missense mutations (e.g., *p.G203R*) typically result in gain-of-function abnormalities, enhancing G*α*o dissociation from G*βγ* and constitutively activating downstream cAMP/PKA and MAPK pathways, thereby causing abnormal neurotransmitter release and epilepsy (6). Although these two mechanisms involve distinct molecular pathways (*NDE1* in neural circuit development, G*α*o in synaptic plasticity), both lead to circuit abnormalities. Their combined effect may be additive or synergistic, worsening developmental encephalopathy and leading to early-onset drug-resistant epilepsy. Additionally, this microdeletion also involves *MYH11*, causing severe functional gastrointestinal obstruction that impairs nutrient absorption and antiepileptic drug bioavailability, further complicating seizure control. In summary, while no direct interaction between the two genetic alterations has been demonstrated, they may exert synergistic effects by jointly affecting neuronal development, synaptic plasticity, and neurotransmitter regulation, thus explaining the patient's severe epilepsy and gastrointestinal dysmotility.

The coexistence of the *GNAO1 p.G203R* variant and the 16p13.11 microdeletion in this infant may explain the drug-resistant epilepsy, reflecting the complex genetic background. Literature indicates that the treatment response for *GNAO1*-related epilepsy, particularly with the *p.G203R* variant, varies substantially, with an overall response rate of approximately 50% (9). Some patients have shown favorable responses to levetiracetam, topiramate, vigabatrin (24), and, more recently, perampanel. The efficacy of the ketogenic diet is inconsistent, and studies in mouse models suggest the potential therapeutic value of zinc supplementation (10, 25–27). Epilepsy associated with the 16p13.11 microdeletion exhibits significant phenotypic heterogeneity and requires tailored, symptomatic management. The infant's treatment course exemplifies this unpredictability. Sequential trials of levetiracetam, adrenocorticotropic hormone, valproate, and oxcarbazepine were undertaken. Transient seizure control and developmental progress occurred only during the postoperative period with combined valproate, but focal seizures subsequently recurred and intensified. It is notable that despite therapeutic doses of valproate, the infant consistently exhibited subtherapeutic serum levels. This was ultimately attributed to drug leakage from a prolapsed intestinal stoma, suggesting that severe functional gastrointestinal obstruction may directly impair drug absorption and contribute to poor seizure control. The EEG evolution—from a burst-suppression pattern to multifocal discharges and focal seizures—reflects the progressive nature of the epileptic encephalopathy. Our study is limited by a short follow-up period and unconfirmed mosaicism. Future studies with larger cohorts are needed to clarify the genotype-phenotype correlations and optimize management strategies for such combined genetic variants.

## Conclusion

We report the first case of an infant with concurrent pathogenic variants in *GNAO1* and a 16p13.11 microdeletion. The early-onset refractory epilepsy suggests a synergistic effect between these two genetic defects. *GNAO1* variants (e.g., *p.G203R*) directly disrupt G protein signaling, altering neuronal excitability. In contrast, the 16p13.11 microdeletion is an independent risk factor for neurodevelopmental disorders. Moreover, the associated severe functional gastrointestinal obstruction and its complications likely exacerbated the epilepsy by impairing the absorption of both nutrients and anti-seizure medications. This case provides insight into how point mutations and copy number variations interact to shape a complex phenotype, with implications for future genetic counseling.

## Data Availability

The datasets presented in this article are not readily available because of ethical and privacy restrictions. Requests to access the datasets should be directed to the corresponding author.

## References

[1] Scheffer IE Berkovic S Capovilla G Connolly MB French J Guilhoto L. ILAE Classification of the epilepsies: position paper of the ILAE commission for classification and terminology. Epilepsia. (2017) 58(4):512–21. 10.1111/epi.13709 28276062 PMC5386840

[2] Nakamura K Kodera H Akita T Shiina M Kato M Hoshino H. *De Novo* mutations in *GNAO1*, encoding a Galphao subunit of heterotrimeric G proteins, cause epileptic encephalopathy. Am J Hum Genet. (2013) 93(3):496–505. 10.1016/j.ajhg.2013.07.014 23993195 PMC3769919

[3] Kulkarni N Tang S Bhardwaj R Bernes S Grebe TA. Progressive movement disorder in brothers carrying a *GNAO1* mutation responsive to deep brain stimulation. J Child Neurol. (2016) 31(2):211–4. 10.1177/0883073815587945 26060304

[4] Olson H Shen Y Avallone J Sheidley BR Pinsky R Bergin AM. Copy number variation plays an important role in clinical epilepsy. Ann Neurol. (2014) 75(6):943–58. 10.1002/ana.24178 24811917 PMC4487364

[5] Palumbi R Ponzi E Micella S Pascali M Bucci R Gentile M. Clinical phenotype of the 16p.13.11 microdeletion: a case report with a mini review of the literature. Front Genet. (2024) 15:1429185. 10.3389/fgene.2024.1429185 39221225 PMC11361943

[6] Feng H Khalil S Neubig RR Sidiropoulos C. A mechanistic review on *GNAO1*-associated movement disorder. Neurobiol Dis. (2018) 116:131–41. 10.1016/j.nbd.2018.05.005 29758257

[7] Lasa-Aranzasti A Larasati YA Da Silva Cardoso J Solis GP Koval A Cazurro-Gutiérrez A. Clinical and molecular profiling in *GNAO1* permits phenotype-genotype correlation. Mov Disord. (2024) 39(9):1578–91. 10.1002/mds.29881 38881224

[8] Arya R Spaeth C Gilbert DL Leach JL Holland KD. *GNAO1*-associated Epileptic encephalopathy and movement disorders: c.607G > A variant represents a probable mutation hotspot with a distinct phenotype. Epileptic Disord. (2017) 19(1):67–75. 10.1684/epd.2017.0888 28202424

[9] Sáez González M Kloosterhuis K Van De Pol L Baas F Robertson SP. Phenotypic diversity in *GNAO1* patients: a comprehensive overview of variants and phenotypes. Hum Mutat. (2023) 2023:6628283. 10.1155/2023/6628283 40225165 PMC11919132

[10] JoJo Yang Q Porter BE Axeen ET. *GNAO1*-related Neurodevelopmental disorder: literature review and caregiver survey. Epilepsy Behav Rep. (2023) 21:100582. 10.1016/j.ebr.2022.100582 36654732 PMC9841045

[11] Kelly MK Park M Mihalek I Rochtus A Gramm M Pérez-Palma E. Spectrum of neurodevelopmental disease associated with the GNAO1 guanosine triphosphate-binding region. Epilepsia. (2019) 60(3):406–18. 10.1111/epi.14653 30682224 PMC6452443

[12] Kim SY Shim YK Ko YJ Park S Jang SS Lim BC. Spectrum of movement disorders in GNAO1 encephalopathy: in-depth phenotyping and case-by-case analysis. Orphanet J Rare Dis. (2020) 15(1):343. 10.1186/s13023-020-01594-3 33298085 PMC7724837

[13] Feng H Larrivee CL Demireva EY Xie H Leipprandt JR Neubig RR. Mouse models of GNAO1-associated movement disorder: allele- and sex-specific differences in phenotypes. PLoS One. (2019) 14(1):e0211066. 10.1371/journal.pone.0211066 30682176 PMC6347370

[14] Larasati YA Savitsky M Koval A Solis GP Valnohova J Katanaev VL. Restoration of the GTPase activity and cellular interactions of Galpha(o) mutants by Zn(2+) in *GNAO1* encephalopathy models. Sci Adv. (2022) 8(40): eabn9350. 10.1126/sciadv.abn9350 36206333 PMC9544338

[15] Luo X Wu L Song J Xu J Huang R Niu H. Prenatal diagnosis, ultrasound findings, and follow-up evaluation of 16p13.11 deletion and duplication syndromes: preliminary assessment of fetal genotype-phenotype. J Clin Lab Anal. (2025) 39(13):e70051. 10.1002/jcla.70051 40534548 PMC12217649

[16] Gavrilovici C Jiang Y Kiroski I Sterley T-L Vandal M Bains J. Behavioral deficits in mice with postnatal disruption of Ndel1 in forebrain excitatory neurons: implications for epilepsy and neuropsychiatric disorders. Cerebral cortex Communications. (2021) 2(1):tgaa096. 10.1093/texcom/tgaa096 33615226 PMC7876307

[17] Billon C Piccoli GB De Sainte Agathe J-M Stoeva R Derive N Heidet L. Genome-wide analysis identifies MYH11 compound heterozygous variants leading to visceral myopathy corresponding to late-onset form of megacystis-microcolon-intestinal hypoperistalsis syndrome. Mol Genet Genomics. (2024) 299(1):44. 10.1007/s00438-024-02136-3 38625590

[18] Liu J Bai Y Feng Y Liu X Pang B Zhang S. ABCC1 Deficiency potentiated noise-induced hearing loss in mice by impairing cochlear antioxidant capacity. Redox Biol. (2024) 74:103218. 10.1016/j.redox.2024.103218 38870779 PMC11225891

[19] Li M Mei L He C Chen H Cai X Liu Y. Extrusion pump ABCC1 was first linked with nonsyndromic hearing loss in humans by stepwise genetic analysis. Genet Med. (2019) 21(12):2744–54. 10.1038/s41436-019-0594-y 31273342

[20] Nollet L Campens L De Zaeytijd J Leroy B Hemelsoet D Coucke PJ. Clinical and subclinical findings in heterozygous ABCC6 carriers: results from a Belgian cohort and clinical practice guidelines. J Med Genet. (2022) 59(5):496–504. 10.1136/jmedgenet-2020-107565 33820832

[21] Cai M Lin N Guo N Huang H Fan X Fu M. Molecular genetic and clinical characteristics of fetuses with chromosome 16 short-arm microdeletions/microduplications. J Clin Lab Anal. (2024) 38(24):e25132. 10.1002/jcla.25132 39665492 PMC11659735

[22] Miteff CI Smith RL Bain NL Subramanian G Brown JE Kamien B. 16p13.11 Microdeletion in a patient with hemiconvulsion-hemiplegia-epilepsy syndrome: a case report. J Child Neurol. (2015) 30(1):83–6. 10.1177/0883073813516382 24453159

[23] Cai M Que Y Chen X Chen Y Liang B Huang H. 16p13.11 Microdeletion/microduplication in fetuses: investigation of associated ultrasound phenotypes, genetic anomalies, and pregnancy outcome follow-up. BMC Pregnancy Childbirth. (2022) 22(1):913. 10.1186/s12884-022-05267-w 36476185 PMC9727942

[24] Yang X Niu X Yang Y Cheng M Zhang J Chen J. Phenotypes of GNAO1 variants in a Chinese cohort. Front Neurol. (2021) 12:662162. 10.3389/fneur.2021.662162 34122306 PMC8193119

[25] Marcé-Grau A Dalton J López-Pisón J García-Jiménez MC Monge-Galindo L Cuenca-León E. *GNAO1* Encephalopathy: further delineation of a severe neurodevelopmental syndrome affecting females. Orphanet J Rare Dis. (2016) 11:38. 10.1186/s13023-016-0416-0 27072799 PMC4830060

[26] Nissenkorn A Kluger G Schubert-Bast S Bayat A Bobylova M Bonanni P. Perampanel as precision therapy in rare genetic epilepsies. Epilepsia. (2023) 64(4):866–74. 10.1111/epi.17530 36734057

[27] Larasati YA Thiel M Koval A Silachev DN Koy A Katanaev VL. Zinc for GNAO1 encephalopathy: preclinical profiling and a clinical case. Med. (2025) 6(1):100495. 10.1016/j.medj.2024.07.023 39153472

